# Pulmonary hypertension in lymphangioleiomyomatosis: prevalence, severity and the role of carbon monoxide diffusion capacity as a screening method

**DOI:** 10.1186/s13023-017-0626-0

**Published:** 2017-04-20

**Authors:** Carolina S. G. Freitas, Bruno G. Baldi, Carlos Jardim, Mariana S. Araujo, Juliana Barbosa Sobral, Gláucia I. Heiden, Ronaldo A. Kairalla, Rogério Souza, Carlos R. R. Carvalho

**Affiliations:** 1 Pulmonary Division, Heart Institute (InCor), University of São Paulo Medical School, Av Dr Enéas de Carvalho Aguiar, 44, 5° andar – sala 1, São Paulo, 05403-900 Brazil; 2 Pulmonology Department, Federal University of Paraná, Curitiba, Brazil; 3 Echocardiography Laboratory, Radiology Institute (InRad), University of São Paulo Medical School, São Paulo, Brazil

**Keywords:** Echocardiography, Lymphangioleiomyomatosis, Prevalence, Pulmonary hypertension, Right heart catheterisation

## Abstract

**Background:**

Lymphangioleiomyomatosis (LAM) is included within group 5 of the current PH classification (unclear multifactorial mechanisms). However, data regarding the occurrence of PH in LAM are scarce. The aims of the study were to describe the prevalence and characteristics of PH in a large cohort of LAM patients with different levels of severity, and to evaluate the role of echocardiography and carbon monoxide diffusion capacity (DL_CO_) as screening methods for PH in LAM.

**Methods:**

One hundred five LAM patients underwent transthoracic echocardiography, pulmonary function tests (PFTs) and 6-min walk test (6MWT). Patients with a suspicion of PH on echocardiography, defined by the presence of estimated systolic pulmonary artery pressure (PAP) over 35 mmHg or PFT showing DLco below 40% of the predicted value, underwent right heart catheterisation to confirm the diagnosis of PH.

**Results:**

Eight patients (7.6%) had PH confirmed on right heart catheterisation, six patients (5.7%) had a pre-capillary pattern and two patients (1.9%) had a post-capillary profile. Only one patient (1%) had mean PAP over 35 mmHg. Patients with PH had lower FEV_1_ and DL_CO_ in PFTs and greater oxygen desaturation and dyspnea intensity during 6MWT compared with those without PH. In 63% of the patients with confirmed PH, the right heart catheterisation was performed based only on DL_CO_ result.

**Conclusions:**

The prevalence of PH is low in LAM patients. Pulmonary hypertension in LAM is typically mild and significantly associated with pulmonary parenchymal involvement. Carbon monoxide diffusion capacity significantly improved the identification of PH in LAM patients.

## Background

Lymphangioleiomyomatosis (LAM) is a rare low-grade neoplasm characterised by proliferation of atypical muscle cells (LAM cells) predominantly around airways, blood vessels and lymphatics, leading to the development of diffuse pulmonary cysts [1–3]. Clinically, it is characterised by progressive dyspnea, recurrent spontaneous pneumothorax, dry cough, hemoptysis and chylothorax, and by extrapulmonary manifestations, such as renal angiomyolipoma and lymphangioleiomyomas [1, 4–6]. Dyspnea and lower exercise capacity may be associated with several factors, such as dynamic hyperinflation, worsening gas exchange and, potentially, pulmonary hypertension (PH). Hypoxemia may occur at rest, during exercise or even during sleep, mainly in those patients with a higher degree of impairment in pulmonary function tests (PFTs) [5, 7, 8].

Pulmonary hypertension is a known complication of LAM, classified among other diseases with unclear multifactorial mechanisms within group 5 of the current PH classification [9]. Several pathophysiological processes might be implicated in the development of PH in LAM. Dysregulation of the mammalian target of rapamycin pathway, a major factor associated with the atypical proliferation of LAM cells, could be related to endothelial dysfunction; furthermore, chronic hypoxic vasoconstriction and even pulmonary artery wall infiltration by LAM cells could also contribute to the increase in pulmonary vascular resistance [10–13].

Nevertheless, data regarding PH in LAM are still scarce. Previous studies evaluating the association of PH and LAM included only patients with impaired lung function and/or were based solely on echocardiographic evaluation to determine the prevalence of PH. To our knowledge, the prevalence and characterisation of PH in patients with different levels of disease severity, including those with normal lung function, have not been completely determined in LAM. The aims of our study are to describe the prevalence and characteristics of PH, and to evaluate the role of echocardiography and carbon monoxide diffusion capacity (DL_CO_) in predicting the presence of PH in a large cohort of patients with LAM followed in a national reference centre.

## Methods

### Study design and participants

This was a cross-sectional, single-centre study conducted at a national reference centre in São Paulo, Brazil. All patients with LAM attending the outpatient clinic of the Pulmonary Division of Hospital das Clínicas, University of São Paulo, were evaluated for inclusion in the study. Patients were required to meet the following criteria: definitive diagnosis of LAM according to European Respiratory Society, American Thoracic Society and Brazilian Thoracic Society guidelines, and clinical stability, defined as the absence of exacerbations for a minimum of 6 weeks [1, 6, 14]. Patients who underwent lung transplant were not enrolled in the study. The protocol was approved by the local research ethics committee, and all patients provided written informed consent before enrollment (protocol number 759.676). All patients performed PFTs, echocardiogram and six-minute walk test (6MWT) at the baseline evaluation; when indicated, right heart catheterisation was performed within 30 days of the initial visit.

### Measurements

#### Pulmonary function tests

All measurements were obtained based on the recommended standards [15–17]. Spirometry was performed using a calibrated pneumotachograph (Medical Graphics Corporation, St, Paul, MN), and lung volumes and DL_CO_ measurements were obtained with a body plethysmograph (Elite Dx, Elite Series; Medical Graphics Corporation). The following variables were obtained: forced vital capacity (FVC), forced expiratory volume in the first second (FEV_1_), total lung capacity (TLC), residual volume (RV) and DL_CO_. Predicted values were derived from the Brazilian population [18–20].

#### Transthoracic echocardiography

All patients underwent two-dimensional transthoracic Doppler echocardiography using IE 33 equipment, Philips Medical Systems, Botthel, USA, to evaluate the following variables: tricuspid regurgitation jet velocity, estimated systolic pulmonary artery pressure (PAP), obtained from the tricuspid regurgitation jet velocity and the inferior vena cava collapsibility index; and left ventricular ejection fraction [21, 22].

#### Six-minute walk test

Patients with LAM performed the 6MWT according to the American Thoracic Society guidelines [23]. Heart rate (HR) and oxygen saturation (SpO_2_) were measured at rest, every minute, and at the end of exercise. Breathlessness was evaluated using a modified Borg scale before and at the end of exercise.

### Right heart catheterisation

All patients with estimated systolic PAP > 35 mmHg at echocardiography and/or DLco < 40% of the predicted value during the PFT, underwent right heart catheterisation to confirm the diagnosis of PH [24–27].

The measurements from the right heart catheterisation were obtained using a pulmonary artery catheter 7 F inserted through the jugular vein for diagnostic evaluation of potential patients with PH [28, 29]. The following variables were recorded: cardiac output (CO), mean pulmonary artery pressure (mPAP), pulmonary artery occlusion pressure (PAOP) and pulmonary vascular resistance (PVR). Cardiac output was measured by thermodilution, considering the average of three consecutive measurements with a maximum variation of 10% among them. Pulmonary hypertension was defined by the presence of mean pulmonary artery pressure ≥ 25 mmHg [9]. Patients with PH were also divided according to the PAOP level into pre-capillary PH (when PAOP ≤ 15 mmHg) or post-capillary PH (when PAOP > 15 mmHg) [30, 31].

### Statistical analysis

Continuous variables are reported as the mean ± SD for those with normal distribution or median (interquartile range) for those with non-normal distribution, whereas categorical variables are presented as proportions. The prevalence of PH is reported as proportion with 95% confidence interval. Unpaired t-tests or Mann-Whitney U test was used for comparison of continuous variables. Categorical variables were compared using the Chi-squared test. Differences were considered significant if *P <* 0.05. Data were analysed with SigmaStat version 3.5 (Systat Software, Inc; San Jose, California).

## Results

**Fig. 1:**
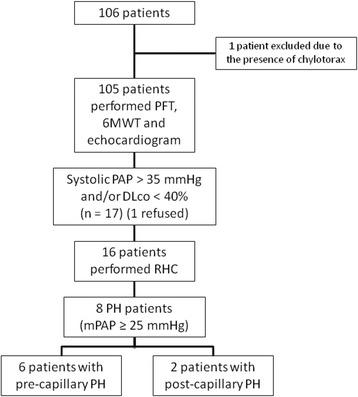
Patient disposition. Definition of abbreviations: 6MWT: six-minute walk test; DL_CO_: lung diffusing capacity for carbon monoxide; mPAP: mean pulmonary arterial pressure; PAP: pulmonary arterial pressure; PFT: pulmonary function tests; PH: pulmonary hypertension; RHC: right heart catheterisation

One hundred six patients with LAM were followed in our outpatient clinic between January 2014 and July 2016; one patient was excluded due to the presence of a large chylothorax, therefore, 105 patients with LAM were enrolled in the study (Fig. 1). Patients had a mean age of 41 ± 13 years and a median time from diagnosis of 5 years (IQR 1 to 9 years). Eighteen patients (17%) had tuberous sclerosis (Table 1).

**Table 1:** Clinical, functional and echocardiographic characteristics (*n* = 105)

Clinical variables	
Age (years)	41 ± 13
Time from LAM diagnosis (years, IQR)	5 (1 – 9)
Smoker (former or current) (n, %)	10 (9.5%)
BMI > 35 kg.m^−2^ (n, %)	2 (2%)
Tuberous sclerosis (n, %)	18 (17%)
Renal angiomyolipoma (n, %)	48 (45%)
Dyspnea (n, %)	74 (70%)
Pneumothorax (n, %)	48 (46%)
Hemoptysis (n, %)	14 (13%)
Use of sirolimus (n, %)	34 (32%)
Duration of use of sirolimus (months)	27 ± 18
Pulmonary function tests	
FEV_1_ (L; % predicted)	2.08 ± 0.72 L; 73 ± 24%
FVC (L; % predicted)	3.01 ± 0.73 L; 86 ± 19%
FEV_1_/FVC	0.68 ± 0.17
RV (L; % predicted)	138 ± 57%
TLC (L; % predicted)	5.08 ± 1.02 L; 103 ± 18%
RV/TLC	0.43 ± 0.15
DLco (mL/min/mmHg; % predicted)	16.7 ± 7.1; 68 ± 28%
Six-minute walk test	
Distance (m; % predicted)	480 ± 114 m; 82 ± 19%
Minimum SpO_2_ (%)	90 ± 8
Change in SpO_2_ (%)	7 ± 5
Peak HR, beats/min	115 ± 19
Final Borg dyspnea score (IQR)	2 (0 – 5)
Final Borg leg discomfort score (IQR)	1 (0 – 3)
Transthoracic echocardiography	
Estimated systolic PAP (mmHg)	27 ± 6
Left ventricular ejection fraction (%)	67 ± 2

Values are the mean ± SD, median (interquartile range) or percentage

*Definition of abbreviations*: *BMI* body mass index, *DL*_*CO*_ lung diffusing capacity for carbon monoxide, *FEV*_*1*_ forced expiratory volume in the first second, *FVC* forced vital capacity, *HR* heart rate, *LAM* lymphangioleiomyomatosis, *PAP* pulmonary arterial pressure, *RV* residual volume, *SpO*_*2*_ oxygen saturation, *TLC* total lung capacity

All patients performed PFTs, 6MWT and echocardiography (Table 1). With respect to the PFTs, FEV_1_ was 2.08 ± 0.72 L (73 ± 24% of predicted value), whereas DL_CO_ was 16.7 ± 7.1 mL/min/mmHg (68 ± 28% of predicted value). Fifty-five patients (52%) presented DL_CO_ below 75%, whereas 14 patients (13%) had DL_CO_ below 40%.

The mean distance walked during the 6MWT was 480 ± 114 m (82 ± 19% of predicted values), whereas the reduction in the SpO_2_ and the minimum SpO_2_ were, respectively, 7 ± 5% and 90 ± 8%. The median Borg dyspnea score at the end of the 6MWT was 2 (IQR 0 to 5).

Based on echocardiography results, estimated systolic PAP was 27 ± 6 mmHg and left ventricular ejection fraction was 67 ± 2%. Six (5.7%) patients had estimated systolic PAP over 35 mmHg.

Of the 105 patients included, 16 patients underwent right heart catheterisation based on DLco and/or echocardiography: two patients had only estimated systolic PAP over 35 mmHg, 11 patients had only DL_CO_ below 40%, and three patients presented both abnormalities. One patient with elevated systolic PAP refused to undergo the procedure. Eight patients (7.6%; 95% CI: 4–14%) had PH confirmed during the right heart catheterisation; six patients (5.7%; 95% CI 2.6–11.9%) presented a pre-capillary pattern and 2 patients (1.9%; 95% CI 0.5 - 6.7%) with a post-capillary profile. Nonetheless, only one patient (1%; 95% CI 0.2–5.2%) had a mean PAP over 35 mmHg, with a post-capillary pattern. In five patients (63%) with confirmed PH, the right heart catheterisation was performed based only on DL_CO_ results.

### Comparison between PH and non-PH groups

**Fig. 2:**
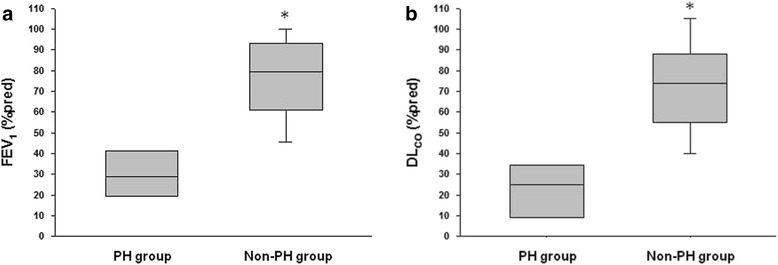
Comparison of FEV_1_ and DL_CO_ between PH *vs.* non-PH groups. Definition of abbreviations: DL_CO_: lung diffusing capacity for carbon monoxide; FEV_1_: forced expiratory volume in the first second; PH: pulmonary hypertension. *p < 0.001. Box plots show the quartiles (box limits), the 10th and 90th percentiles (error bars) and the median (line)

When comparing patients with and without PH, there was no significant difference in terms of age and time from diagnosis. Patients with PH had a higher frequency of use of sirolimus, worse functional impairment, characterised by lower FEV_1_ and DL_CO_, and diminished exercise performance, greater oxygen desaturation and higher dyspnea intensity during 6MWT, compared with the non-PH group (Table 2 and Fig. 2).

**Table 2:** Clinical, functional and echocardiographic variables, and data obtained from right heart catheterisation: comparison between PH and non-PH groups

Clinical, functional and echocardiographic variables	PH (*n* = 8)	Non-PH (*n* = 97)	P
Age (years)	44 ± 6	41 ± 13	0.60
Time from LAM diagnosis (years, IQR)	2 (1.5–11.5)	5 (1–9)	0.75
Use of sirolimus (n, %)	7 (87%)	27 (28%)	0.002
Duration of use of sirolimus (months)	23 ± 7	28 ± 20	0.57
FEV_1_ (% predicted)	33 ± 18	76 ± 21	<0.001
DL_CO_ (% predicted)	24 ± 14	72 ± 26	< 0.001
6MWD (m)	371 ± 113	488 ± 110	0.01
Minimum SpO_2_	82 ± 6	91 ± 8	0.01
Final Borg dyspnea score	5 (5–7)	2 (0–5)	0.048
Estimated systolic PAP	38 ± 7	26 ± 5	< 0.001
Right heart catheterisation	PH (*n* = 8)	Non-PH (*n* = 8)	
mPAP (mmHg)	29 ± 5	21 ± 2	<0.001
PAOP (mmHg)	14 ± 4	10 ± 5	0.11
CO (L/min)	4.8 ± 1.2	5.0 ± 0.6	0.70
PVR (IU)	3.4 ± 1.2	2.3 ± 0.8	0.06

Values are the mean ± SD or median (interquartile range)

*Definition of abbreviations*: *6MWD* six-minute walk distance, *CO* cardiac output, *DL*_*CO*_ lung diffusing capacity for carbon monoxide, *FEV*_*1*_ forced expiratory volume in the first second, *LAM* lymphangioleiomyomatosis, *mPAP* mean pulmonary arterial pressure, *PH* pulmonary hypertension, *PAOP* pulmonary artery occlusion pressure, *PAP* pulmonary arterial pressure, *PVR* pulmonary vascular resistance, *SpO*_*2*_ oxygen saturation

We also compared data obtained from right heart catheterisation between PH and non-PH groups (Table 2); as expected, mPAP was higher in the PH group but with similar levels of CO.

## Discussion

To our knowledge, this is the first study that has evaluated the prevalence of PH in patients with LAM with different severity levels, including patients with normal PFTs, comparing the clinical, functional and echocardiographic characteristics, and variables obtained from right heart catheterisation between those with and without PH. The main findings of this study include the following: 1) The prevalence of PH was low in patients with LAM; 2) PH is of mild severity in LAM and may be pre- or post-capillary; 3) PH was associated with greater impairment of pulmonary function, which suggests that the elevation in PAP is likely associated with the degree of pulmonary parenchymal involvement; 4) Patients with PH had lower exercise performance and greater dyspnea intensity and desaturation during 6MWT; and 5) Reduced DL_CO_ added sensitivity in predicting PH in LAM.

Cottin et al. described the findings of 20 patients with LAM and with pre-capillary PH. However, this previous study was not designed to define the prevalence of PH in LAM, because echocardiography was performed at the discretion of the physicians and the routine was to perform it in those patients with impaired lung function. In addition, as DL_CO_ was not used as an associated tool for screening of PH, the number of patients with confirmed PH may have been underestimated. We performed echocardiography even in patients with normal PFTs, which resulted in a more precise prevalence of PH. Our study, by prospectively evaluating a large cohort of LAM patients, determined a prevalence of pre-capillary PH of 5.7%. It is noteworthy that no patients with pre-capillary PH presented with mean PAP > 35 mmHg, a threshold suggesting PH to be important in the setting of lung diseases. Patients with PH presented with worse pulmonary function and exercise capacity despite preserved CO [11].

Taveira-DaSilva et al. evaluated 95 patients with echocardiography and identified elevation of systolic PAP at rest in less than 10% of patients with LAM, with only mild increase in PAP. The results obtained in this previous study suggested hypoxemia as an important mechanism for the increase in pulmonary vascular resistance in LAM. However, the authors did not perform invasive hemodynamic evaluation to confirm the presence of PH and there was a failure to estimate PAP on echocardiography in approximately 20% of the patients [10]. Our study reinforced that the use of echocardiography as the single tool for the screening of PH in LAM might be of limited sensitivity, underestimating the prevalence of PH, due to the lack of an adequate chest window in a significant proportion of patients, potentially as a consequence of lung hyperinflation. Therefore, we decided to include DL_CO_ as a secondary screening tool to determine the need for invasive hemodynamic evaluation in order to increase the sensitivity of screening. Most patients with confirmed PH during right heart catheterisation were referred to the test only because of the DL_CO_ levels (five out of eight patients, 63%), raising the question about the appropriateness of echocardiography as the main screening tool in patients with parenchymal lung diseases, as suggested in the current guidelines [32].

As there are only few studies of PH in LAM, we chose the methods of screening of PH in our study based on data obtained in studies that evaluated PH in other diseases, such as chronic obstructive pulmonary disease, idiopathic pulmonary fibrosis (IPF), systemic sclerosis, sickle cell disease and schistosomiasis. Echocardiography is a widespread screening method for PH, established in many clinical scenarios, and the presence of estimated systolic PAP > 35 mmHg at echocardiography has mostly acceptable sensitivity for the indication of right heart catheterisation [21, 22, 33–35]. In addition, other studies have shown that low DL_CO_ may predict the presence of PH [26–28]. In patients with IPF the presence of low DL_CO_ added sensitivity to the echocardiographic findings in the screening for PH and is associated with a higher risk of PH [25–27]. Nathan et al. showed that DL_CO_ below 40% of the predicted value had high sensitivity to predict the diagnosis of PH [25]. Raghu et al. recently showed that patients with IPF and PH had lower DL_CO_ than those without PH (39% *vs.* 44%, respectively, *P* = 0.002) [27]. In the DETECT study, 466 patients with systemic sclerosis underwent right heart catheterisation to confirm the diagnosis of PH and those with PH had lower DL_CO_ than those without PH [24]. Therefore, based on our findings and in previous studies, we considered that DL_CO_ could be added as a reasonable screening method of PH in patients with parenchymal lung diseases.

In patients with systemic sclerosis, the presence of mild elevations in mPAP is associated with a higher risk of future development of PH [36]; however, the significance of such finding in other clinical scenarios is still unknown. The non-PH group in our study presented mPAP of 21 ± 2 mmHg; close follow-up of our patients that presented this haemodynamic profile will determine the relevance of this finding in predicting the development of PH in LAM patients.

Previous reports of LAM cells involving pulmonary vessels and vascular remodeling could suggest a direct pulmonary vascular involvement as the cause of PH in LAM [11, 12]. However, these previous findings were observed mostly in patients with severe parenchymal lung disease, and our study also showed that PH was associated with a degree of parenchymal involvement in LAM, as in other lung diseases, which explains the higher prevalence of use of sirolimus in those with PH. This suggests that PH associated with LAM should be considered among the other parenchymal lung diseases, as part of the group 3 of the current PH classification, instead of its current position in group 5, which comprises PH-associated diseases with unclear multifactorial mechanisms [9].

Despite our important findings, this study has limitations that should be acknowledged. Although this was a single-reference-centre study, it included all patients followed in our centre, which is a national reference centre receiving patients from all over the country. Therefore, it is reasonable to generalise the results of our study for all patients with LAM in Brazil. Another limitation is the use of echocardiographic and pulmonary function criteria based on other diseases, such as IPF and scleroderma; however, this was the only way to question the sensitivity of echocardiography as a single screening method without submitting all patients to right heart catheterisation, which would not be acceptable or ethical in this setting. In this context, although our strategy of combining DL_CO_ below 40% of the predicted value and an echocardiographic criteria as screening tools has added sensitivity in predicting PH in LAM, we cannot completely exclude the presence of mild PH in those with DL_CO_ equal or greater than 40% of the predicted value, since right heart catheterisation was not performed in all patients.

## Conclusions

Our study demonstrated that the prevalence of PH in a large cohort of LAM patients with different levels of severity is low and with mild hemodynamic impairment. Furthermore, PH was associated with more pronounced pulmonary function impairment, suggesting that LAM associated PH should be better considered among the other parenchymal lung diseases associated with the development of PH, which compose group 3 of the current classification of PH. In addition, DL_CO_ significantly improved the identification of PH in LAM patients.

## Abbreviations

6MWD: Six-minute walk distance
6MWT: Six-minute walk test
BMI: Body mass index
CO: Cardiac output
DL_CO_: Carbon monoxide diffusion capacity
FEV_1_: Forced expiratory volume in the first second
FVC: Forced vital capacity
HR: Heart rate
LAM: Lymphangioleiomyomatosis
mPAP: Mean pulmonary artery pressure
PAOP: Pulmonary artery occlusion pressure
PAP: Pulmonary artery pressure
PFT: Pulmonary function test
PH: Pulmonary hypertension
PVR: Pulmonary vascular resistance
RHC: Right heart catheterisation
RV: Residual volume
SpO_2_: Oxygen saturation
TLC: Total lung capacity
